# GLI1+ cells are a source of repair-supportive mesenchymal cells (RSMCs) during airway epithelial regeneration

**DOI:** 10.1007/s00018-022-04599-2

**Published:** 2022-11-05

**Authors:** Xuran Chu, Arun Lingampally, Alena Moiseenko, Vahid Kheirollahi, Ana Ivonne Vazquez-Armendariz, Janine Koepke, Ali Khadim, Georgios Kiliaris, Mahtab Shahriari Felordi, Mahsa Zabihi, Irina Shalashova, Ioannis Alexopoulos, Stefan Günther, Kevin Lebrigand, Marin Truchi, Andreas Günther, Thomas Braun, Bernard Mari, Christos Samakovlis, Xiaokun Li, Werner Seeger, Susanne Herold, Jin-San Zhang, Saverio Bellusci, Elie El Agha

**Affiliations:** 1 Oujiang Laboratory (Zhejiang Lab for Regenerative Medicine, Vision and Brain Health), School of Pharmaceutical Science, Wenzhou Medical University, Wenzhou, Zhejiang P.R. China; 2 School of Pharmaceutical Sciences, Wenzhou Medical University, Wenzhou, Zhejiang P.R. China; 3 Department of Medicine II, Internal Medicine, Pulmonary and Critical Care, Universities of Giessen and Marburg Lung Center (UGMLC), German Center for Lung Research (DZL), Justus-Liebig University Giessen, Giessen, Germany; 4 Department of Medicine V, Internal Medicine, Infectious Diseases and Infection Control, Universities of Giessen and Marburg Lung Center (UGMLC), German Center for Lung Research (DZL), Justus-Liebig University Giessen, Giessen, Germany; 5 Cardio-Pulmonary Institute (CPI), Giessen, Germany; 6 Institute for Lung Health (ILH), Giessen, Germany; 7 Max Planck Institute for Heart and Lung Research, W.G. Kerckhoff Institute, Bad Nauheim, Germany; 8 Université Côte d’Azur, CNRS, IPMC, Sophia Antipolis, Valbonne, France; 9 Medical Research Center, The First Affiliated Hospital of Wenzhou Medical University, Wenzhou, Zhejiang P.R. China

**Keywords:** Repair-supportive mesenchymal cells (RSMCs), Airway smooth muscle cells (ASMCs), Naphthalene, Airway regeneration, FGF10, GLI1

## Abstract

Repair-supportive mesenchymal cells (RSMCs) have been recently reported in the context of naphthalene (NA)-induced airway injury and regeneration. These cells transiently express smooth muscle actin (*Acta2*) and are enriched with platelet-derived growth factor receptor alpha (*Pdgfra*) and fibroblast growth factor 10 (*Fgf10*) expression. Genetic deletion of *Ctnnb1* (gene coding for beta catenin) or *Fgf10* in these cells using the *Acta2-Cre-ERT2* driver line after injury (defined as NA-Tam condition; Tam refers to tamoxifen) led to impaired repair of the airway epithelium. In this study, we demonstrate that RSMCs are mostly captured using the *Acta2-Cre-ERT2* driver when labeling occurs after (NA-Tam condition) rather than before injury (Tam-NA condition), and that their expansion occurs mostly between days 3 and 7 following NA treatment. Previous studies have shown that lineage-traced peribronchial GLI1+ cells are transiently amplified after NA injury. Here, we report that *Gli1* expression is enriched in RSMCs. Using lineage tracing with *Gli1*^*Cre−ERT2*^ mice combined with genetic inactivation of *Fgf10*, we show that GLI1+ cells with *Fgf10* deletion fail to amplify around the injured airways, thus resulting in impaired airway epithelial repair. Interestingly, *Fgf10* expression is not upregulated in GLI1+ cells following NA treatment, suggesting that epithelial repair is mostly due to the increased number of *Fgf10*-expressing GLI1+ cells. Co-culture of SCGB1A1+ cells with GLI1+ cells isolated from non-injured or injured lungs showed that GLI1+ cells from these two conditions are similarly capable of supporting bronchiolar organoid (or bronchiolosphere) formation. Single-cell RNA sequencing on sorted lineage-labeled cells showed that the RSMC signature resembles that of alveolar fibroblasts. Altogether, our study provides strong evidence for the involvement of mesenchymal progenitors in airway epithelial regeneration and highlights the critical role played by *Fgf10*-expressing GLI1+ cells in this context.

**Supplementary Information:**

The online version contains supplementary material available at 10.1007/s00018-022-04599-2.

## Introduction

The bronchi of the lung represent important interfaces with the outside environment and are as such exposed to many toxins and pathogens. Therefore, the bronchial epithelium undergoes a process of injury repair. The relevant bronchial epithelial cells for the repair process in the bronchi are the non-ciliated secretory cells or club cells [1, 2]. These cells express secretoglobin family 1A member 1 (SCGB1A1, a.k.a. Clara cell 10 kDa secretory protein CC10 or CCSP), and they represent progenitors for both club and ciliated cells [3, 4]. A large subset of club cells expresses high levels of cytochrome P450 family 2 subfamily f polypeptide 2 (CYP2F2), which is involved in the transformation of naphthalene (NA) into trans-1R-hydroxy-2R-glutathionyl-1,2-dihydronaphthalene, a toxic intermediate that leads to the depletion of club cells in the lung [5–7]. In addition, club cells expressing low levels of CYP2F2, called variant club cells, escape the deleterious effect of NA and represent a pool of progenitors. These cells are present at proximity of neuroepithelial bodies (NEBs) and bronchioalveolar duct junctions (BADJs). These cells are critical for the repair process [8–10]. The NA injury model is therefore useful to investigate airway regeneration.

Epithelial–mesenchymal interactions orchestrated by fibroblast growth factors, which have been described in the embryonic lung [11], are also at play after injury in the adult lung. It has been proposed that following NA injury in adult mice, WNT7b expressed by surviving ciliated cells acts on airway smooth muscle cells (ASMCs) leading to the increased expression of *Fgf10* [12]. FGF10 binds to and activates FGFR2b expressed in variant club cells to control their amplification [12]. Leucine-rich repeat-containing G-protein coupled receptor 6 (*Lgr6*)*-*positive cells represent an ASMC subpopulation that enhances repair of the bronchial epithelium following NA administration and also involves a WNT-FGF10 positive feedback loop [13]. Therefore, there is a consensus in the field that ASMCs are a mesenchymal niche for variant club cells following injury.

In a recently published paper, we showed that during lung repair after NA injury, a mesenchymal population of ACTA2+ PDGFRα+ cells emerges in the non-cartilaginous conducting airway niche, which is normally populated by ASMCs [14]. This cell population, which we termed “repair-supportive mesenchymal cells” (RSMCs), is distinct from conventional ASMCs, which have previously been proposed to contribute to epithelial repair [14]. Gene expression analysis on sorted lineage-labeled cells showed that RSMCs express low levels of ASMC marker genes, but high levels of the pro-regenerative marker *Fgf10* [14]. Organoid co-cultures demonstrated an enhanced ability for ACTA2+ cell-derived RSMCs in supporting club cell growth in vitro [14, 15].

In this study, we carried out lineage tracing of ACTA2+ cells captured before (defined as Tam-NA condition) or after NA injury (defined as NA-Tam condition). We also analyzed the contribution of FGF10 derived from the GLI1+ lineage to the repair process. Bronchiolosphere cultures using GLI1+ mesenchymal cells isolated from non-injured or injured lungs co-cultured with SCGB1A1+ cells were also carried out. Our study demonstrates the critical role played by *Fgf10*-expressing GLI1+ cells in the context of repair of the bronchial epithelium.

## Materials and methods

### Mice, tamoxifen administration and naphthalene treatment

*Acta2-Cre-ERT2* mice (STOCK *Tg(Acta2-cre/ERT2)12Pcn*) [16] were kindly provided by Dr. Pierre Chambon (University of Strasbourg, France). *Fgf10*^*flox*^ mice (B6;129-*Fgf10*^*tm1.2Sms*^ /J) [17, 18] were a generous gift from Dr. Suzanne L. Mansour (University of Utah, USA). *tm9 tdTomato*^*flox*^ (hereafter referred to as *tdTomato*^*flox*^) (B6;129S6-*Gt(ROSA)26Sor*^*tm9(CAG−tdTomato)Hze*^ /J, JAX stock number 007905), *tm14 tdTomato*^*flox*^ (B6.Cg-*Gt(ROSA)26Sor*^*tm14(CAG−tdTomato)Hze*^ /J, JAX stock number 007914), *Scgb1a1*^*Cre−ERT*^ (B6N.129S6(Cg)-*Scgb1a1*^*tm1(cre/ERT)Blh*^ /J, JAX stock number 016225) and *Gli1*^*Cre−ERT2*^ (STOCK *Gli1*^*tm3(cre/ERT2)Alj*^ /J, JAX stock number 007913) were purchased from the Jackson laboratory. The *tm14 tdTomato*^*flox*^ line was used in combination with the *Scgb1a1*^*Cre−ERT*^ line, while the *tm9 tdTomato*^*flox*^ line was used in combination with the *Acta2-Cre-ERT2*, *Gli1*^*Cre−ERT2*^ and *Gli1*^*Cre−ERT2*^*; Fgf10*^*flox*^ lines. To induce Cre-ERT2-mediated genetic recombination, *Acta2-Cre-ERT2* and *Gli1*^*Cre−ERT2*^ mice were fed tamoxifen-containing food (0.4 g of tamoxifen per kg of food) (Altromin, Germany). Tamoxifen powder was purchased from Sigma-Aldrich. For naphthalene treatment, 8–12-week-old female mice were used. Naphthalene (Sigma) was dissolved in corn oil (20 mg/mL) and was administered intraperitoneally at a dose of 0,275 mg naphthalene per g of body weight.

### Immunofluorescence

Lungs were flushed by transcardiac perfusion with PBS and then, either fixed with 4% paraformaldehyde for paraffin embedding and 5 μm sectioning using a microtome or inflated with 1.7% low melting agarose for 200 μm sectioning using a vibratome. Immunofluorescence (IF) was performed using monoclonal anti-ACTA2 (Sigma, 1:200), monoclonal anti-SCGB1A1 (Santa Cruz Biotechnology, 1:200), polyclonal anti-KI67 (Thermo Fisher Scientific, 1:200) and polyclonal anti-RFP antibodies (Thermo Fisher Scientific, 1:200; used only when combined with KI67 staining which requires antigen retrieval). Nuclei were stained using DAPI (Life Technologies). Tile scans of thin sections were acquired using EVOS FL2 Imaging System (Thermo Fischer Scientific). Other fluorescent images were acquired using a Leica DM550 B fluorescence microscope, an SP5 or SP8 confocal microscope, both equipped with a white-light laser and hybrid detectors (Leica Microsystems). Where applicable, three-dimensional (3D) reconstruction of z-stacks was performed using LAS X software (version 3.5; Leica Microsystems). For quantification of stained control and experimental lungs, sections from at least 3 independent lungs were analyzed and multiple images were used (*n > *8).

### Flow cytometry analysis and cell sorting

Flow cytometry and cell sorting were carried out as we previously described [14]. In brief, lungs were isolated in Hank’s Balanced Salt Solution (HBSS, Gibco), minced into small pieces and incubated with 0.5% collagenase type IV in HBSS (Life Technologies) at 37 °C for 45 min. Lung homogenates were then passed through 70 and 40 μm cell strainers (BD Biosciences) to obtain single-cell suspensions. Cells were centrifuged at 4 °C at 1000 rpm for 5 min and then resuspended in MACS buffer and stained with anti-PDGFRα (APC-conjugated, 1:100), anti-EpCAM (APC-Cy7-conjugated, 1:100), anti-CD31 (Pacific Blue-conjugated, 1:100) and anti-CD45 (Pacific Blue-conjugated, 1:100) antibodies (all from Biolegend) for 20 min on ice in the dark. Then, cells were washed with MACS buffer. Flow cytometry and cell sorting were done using FACSAria III cell sorter (BD Biosciences).

### Quantitative real-time PCR

For bulk RNA extraction from lungs, accessory lobes were lysed and subjected to RNeasy Mini kit (Qiagen) as we previously described [14]. Extraction of RNA from sorted cells was performed using RNeasy Micro kit (Qiagen). Primers were designed using the Universal Probe Library Assay Design center program (Roche Applied Science), and quantitative real-time PCR (qPCR) was performed using Light Cycler 480 II (Roche Applied Science). Hypoxantine-guanine phosphoribosyltransferase (*Hprt*) was used as a reference gene.

### Single-cell RNA sequencing

Single-cell RNA-seq of tdTomato+ (hereafter referred to as tdTom+) cells isolated from *Acta2-Cre-ERT2; tdTomato*^*flox*^ lungs was performed using the ICELL8 platform (Takara Bio) using the V2 protocol with in-chip preamplification. Samples were presorted into HBSS (Gibco) at a concentration of 20 cells/µL and stained with Hoechst dye (Invitrogen) for 5 min at RT prior to loading onto the MSND dispenser. For each sample, 3 dispenses were made on one 5,184-well chip resulting in 348 and 459 wells containing single cells after selection in CellSelect software (Takara Bio). Selected wells were processed for RT and in-chip preamplification, and cDNA was pooled for final library preparation using standard Nextera protocol (Illumina). The library was checked in Labchip Gx touch 24 (Perkin Elmer) and sequenced on Nextseq500 (Illumina) using V2 chemistry and paired-end setup (11 bp Read1 for cell barcode and 80 bp Read2 for cDNA template sequencing). A total of 537 M reads were obtained.

Single-cell RNA-seq of tdTom+ cells isolated from *Gli1*^*Cre−ERT2*^*; tdTomato*^*flox*^ lungs was carried out using the 10x Genomics Single-Cell 3′ v3.1 RNA-seq kit. Gene expression libraries were prepared according to the manufacturer’s protocol. MULTI-seq barcode libraries were retrieved from the samples, and libraries were prepared independently.

### Single-cell data analysis

ICELL8 platform: cDNA reads were trimmed for poly(A) tails using BBMAP (unpublished, https://jgi.doe.gov/data-and-tools/bbtools/). Reads of length under 50 bases were discarded. The 456 M remaining reads were then aligned using STAR (release v2.4.0a) against *Mus musculus* build mm10 following ENCODE RNA-seq mapping recommendations. STAR indices were generated using Ensembl GTF file (release 83). For counting based on read counts, we used the Dropseq Core Computational Protocol version 1.12 (dropseq.jar) [19]. Gene-to-cell count table matrix was then used for statistical analysis performed within the Seurat package (http://satijalab.org/seurat/) [20]. The pipeline goes through cell and gene filtering, data normalization, then finding the most variable genes to perform principal component analysis (PCA) and t-distributed stochastic neighbor embedding (t-SNE) dimension reduction for visualization purpose. We first created a Seurat object including all cells and all genes present in at least 5 different cells. We then filtered out cells with more than 95% dropouts or less than 50,000 read counts. We also discarded cells with more than 20% of counts from mitochondrial genes or 10% counts from ribosomal genes. We then normalized the count values for the 305 remaining cells to a scaling factor corresponding to the median read counts per cell. We next identified most variable genes by plotting genes into bins based on X-axis (average expression) and Y-axis (dispersion) using cutoffs *X = *3 and *Y = *0.1, identifying 1,212 genes. We then performed a PCA, after scaling and centering of the data across these genes. This ensured robust identification of the primary structures in the data. We identified the first 8 principal components as statistically significant and used it for t-SNE calculation (perplexity = 30). The t-SNE procedure returns a two-dimensional (2D) embedding of single cells utilized for dataset visualization. Cells with similar expression signatures of genes within the variable set, and therefore, similar PC loadings, are most likely to localize near each other in the embedding, and hence, distinct cell types form 2D point clouds across the t-SNE map. Identification of the clusters of cells was determined using the FindClusters Seurat function (resolution = 1). The Seurat FindAllMarkers function was used to identify differentially expressed genes across each cluster (min.pct = 0.25, thresh.use = 0.25).

Chromium 10x platform: Single-cell suspensions were processed using the Chromium Next GEM Single-Cell 3ʹ Reagent Kits v3.1 (10x Genomics). Each sample was run separately on a lane in Chromium controller, and gene expression libraries were prepared according to the manufacturer’s protocol. Library sample index sequences were incorporated as the sample index read and sequenced together on a Nextseq2000 sequencer. Raw reads were aligned against the mouse genome (mm10, ensemble assembly 104) and mapped and counted by StarSolo [21]. Secondary analyses were carried out following the Seurat workflow. We removed 285 cells that did not express more than 1,000 genes, with less than 20,000 detected UMIs or had a mitochondrial content above 15%. Raw counts were normalized and scaled using the SCTransform function. The dimension of the count matrix was reduced by PCA. We used the first 40 principal components to compute the UMAP embedding and construct the nearest neighbor graph. Clusters were identified using a resolution of 0.3 and annotated according to the expression of markers described in the literature [22]. Gene expression exploration and differential analyses were carried out on log-normalized data.

### Bronchiolosphere assay

Sorted mesenchymal cells (tdTom+) from *Gli1*^*Cre−ERT2*^*; tdTomato*^*flox*^ or *Gli1*^*Cre−ERT2*^*; tdTomato*^*flox*^*; Fgf10*^*flox/flox*^ lungs and epithelial cells (tdTom+) from *Scgb1a1*^*CreERT*^*; tdTomato*^*flox*^ lungs or epithelial progenitors from wild-type mice (defined as EpCAM^high^ CD24^low^) were centrifuged, resuspended in cell culture medium (Dulbecco’s Modified Eagle Medium, Life Technologies) and subjected to organoid cultures as we previously described [14, 15, 23]. Cells were incubated under air–liquid conditions at 37 °C with 5% CO_2_ for 2 weeks, and the culture medium was changed 3 times per week.

### Statistical analysis and figure assembly

Quantitative data were assembled and analyzed using GraphPad Prism software (GraphPad Software). These data are presented as average values ± standard error of mean (sem). Student’s t-test was used to compare 2 groups, one-way ANOVA was used to compare 3 or more groups in the presence of 1 variable (e.g., treatment, time or genotype), and two-way ANOVA was used to compare multiple groups in the presence of 2 variables (e.g., treatment and genotype, time and genotype). Differences were considered significant if *P < *0.05. The number of biological replicates is indicated in the corresponding figure legends. Figures were assembled using Adobe Illustrator (Adobe).

## Results

### Mesenchymal cells transiently expressing *Acta2* emerge during epithelial injury and repair

**Fig. 1:**
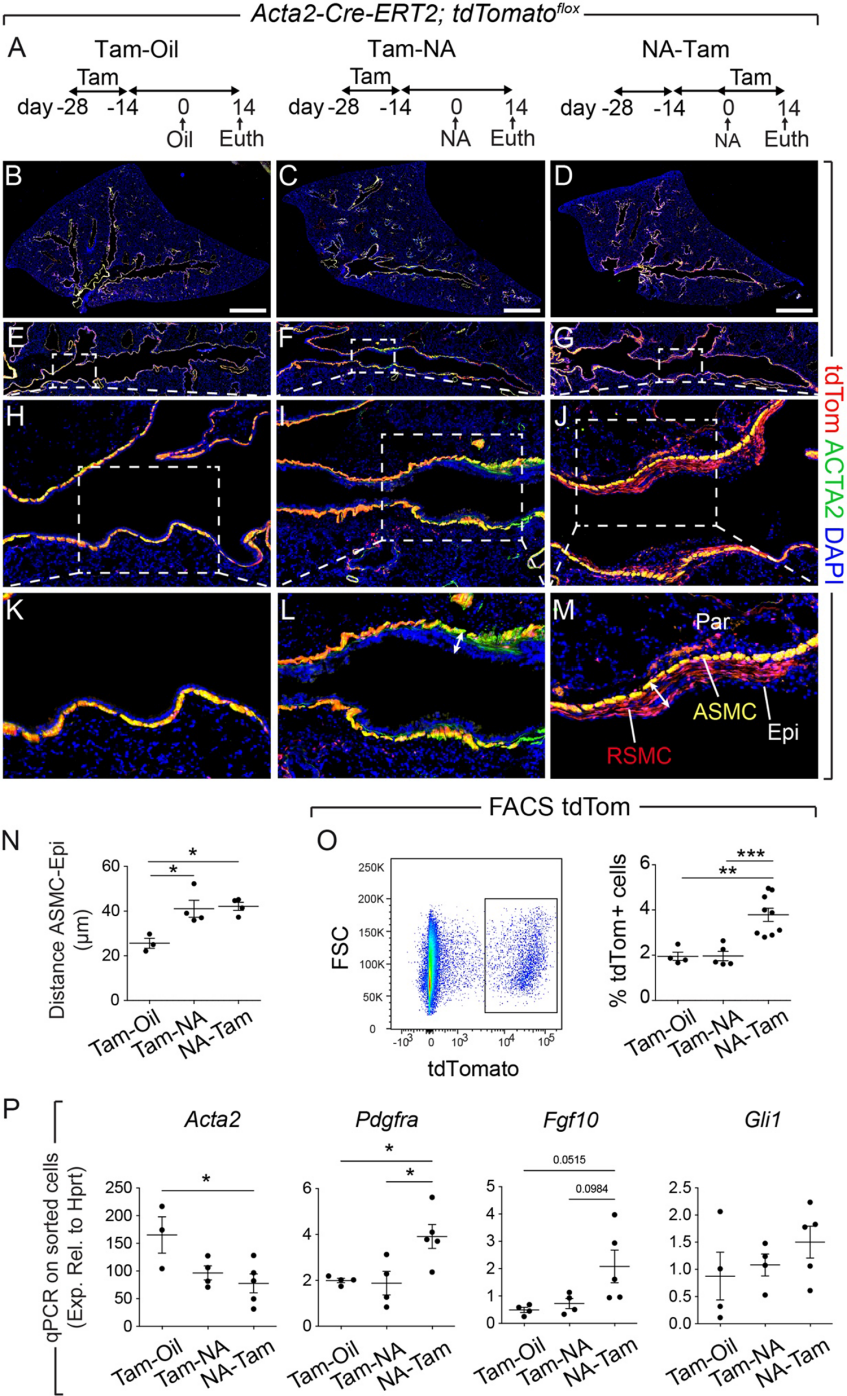
A population of mesenchymal cells transiently expressing *Acta2* is recruited after naphthalene injury. **A** Experimental setup and timeline of tamoxifen and naphthalene/corn oil treatment. Mice were fed tamoxifen-containing food. **B–D** Tile scans of left lung lobes from control oil-treated mice (Tam-Oil), naphthalene-treated mice where pre-existing ACTA2+ cells were labeled (Tam-NA) and naphthalene-treated mice where pre-existing and newly formed ACTA2+ cells were labeled (NA-Tam). Lung sections were stained with anti-ACTA2 antibodies (green), and the endogenous tdTomato signal (red) was detected. **E–G** Zoomed-in images of the conducting airway. **H–J** Zoomed-in images of the boxes in **E**–**G**. **K–M** Zoomed-in images of the boxes in **H–J**. Note the appearance of an additional population of ACTA2− tdTom+ cells (RSMC) in the NA-Tam group (**J**, **M**). **N** Quantification of the distance between ASMC and the epithelium. The double arrows in **L** and **M** illustrate the measured distance. **O** Gating strategy and quantification of tdTom+ cells in Tam-Oil, Tam-NA and NA-Tam lungs at d14. **P** *Acta2, Pdgfra*, *Fgf10* and *Gli1* expression levels analyzed by qPCR using sorted tdTom+ cells from Tam-Oil, Tam-NA and NA-Tam lungs at d14. *ASMC* airway smooth muscle cells, *Epi* epithelium, *FSC* forward scatter, *NA* naphthalene, *Par* parenchyma, *RSMC* repair-supportive mesenchymal cells, *Tam* tamoxifen. Scale bars: (B-D) 1400 μm. (N) *n* = 3 for Tam-Oil, *n* = 4 for Tam-NA and *n* = 4 for NA-Tam; (O) *n* = 4 for Tam-Oil, *n* = 5 for Tam-NA and *n* = 9 for NATam; (P) *n* = 3-4 for Tam-Oil, *n* = 4 for Tam-NA and *n* = 5 for NA-Tam. *P < 0.05, **P < 0.01, ***P < 0.001

In order to analyze the spatial distribution of the ACTA2+ lineage around the injured airways, including our recently reported RSMC population [14], the *Acta2-Cre-ERT2; tdTomato*^*flox*^ lineage-tracing model was employed. Animals were randomly allocated into 3 groups receiving either tamoxifen food prior to corn oil injection (Tam-Oil), tamoxifen food prior to NA injection (Tam-NA) or NA injection followed by tamoxifen food (NA-Tam) (Fig. 1A). Animals were euthanized at day 14 (d14) after NA or Oil injections. Such approach allowed labeling (and visualizing) steady-state ACTA2+ cells, including those around the airways (ASMCs) (Tam-Oil), pre-existing ACTA2+ cells (including ASMCs) during repair after injury (Tam-NA) and total ACTA2+ cells during repair after injury (*bona fide* SMCs and newly recruited ACTA2+ cells; RSMCs) (NA-Tam). While the lineage label, tdTom, colocalized with the ACTA2 stain in the Tam-Oil (Fig. 1B, E, H, K) and Tam-NA groups (Fig. 1C, F, I, L), imaging of NA-Tam lungs revealed, in addition to ACTA2+ tdTom+ cells in the ASMC layer, the presence of ACTA2− tdTom+ cells between the ASMC layer and the airway epithelium, corresponding to RSMCs (Fig. 1D, G, J, M). These cells were clearly distinct from ASMCs and were mostly observed around the proximal lobular airways. They were more abundant around the mainstem bronchus and at the branch points. Quantification of the distance between the ASMC layer and airway epithelium showed significant thickening of the peribronchial region in Tam-NA, similarly to NA-Tam, compared to the Tam-Oil condition (Fig. 1N), confirming that the additional layer of RSMCs also forms in the Tam-NA condition but cannot be visualized due to the timing of tamoxifen administration (before injury).

The number of tdTom+ cells in these three experimental conditions was quantified by flow cytometry (Fig. 1O). Compared to the number of tdTom+ cells in Tam-Oil, our results demonstrate an increase in NA-Tam compared to Tam-Oil and Tam-NA, confirming that additional cells acquire ACTA2 expression after NA injury. We also investigated *Acta2, Pdgfra*, *Fgf10* and *Gli1* expression in sorted tdTom+ cells isolated from Tam-Oil, Tam-NA and NA-Tam lungs. Our results indicate that *Pdgfra* and *Fgf10* expression are indeed upregulated in NA-Tam tdTom+ cells (RSMC-enriched) compared to Tam-Oil or Tam-NA conditions (both SMC-enriched), while *Acta2* expression showed the opposite trend (Fig. 1P). *Gli1* expression showed a trend for upregulation in NA-Tam compared to the other groups (Fig. 1P).

The emergence of tdTom+ RSMCs in NA-Tam, but not Tam-NA, lungs indicated that those cells were descendants of a mesenchymal population that transiently acquired *Acta2* expression after NA injury. To study the dynamics of tdTom+ cells that are labeled after injury, NA-Tam lungs were analyzed by flow cytometry at d3, d7 and d14 (Fig. S1A, B). The results revealed a significant increase in the abundance of tdTom+ cells from d3 to d7 after NA injury (Fig. S1C). Interestingly, such difference in tdTom+ cells was no longer seen between d7 and d14 (Fig. S1C), indicating that RSMCs peaked in terms of abundance at d7.

### Single-cell transcriptomic analysis reveals a mesenchymal niche that is associated with epithelial regeneration

**Fig. 2:**
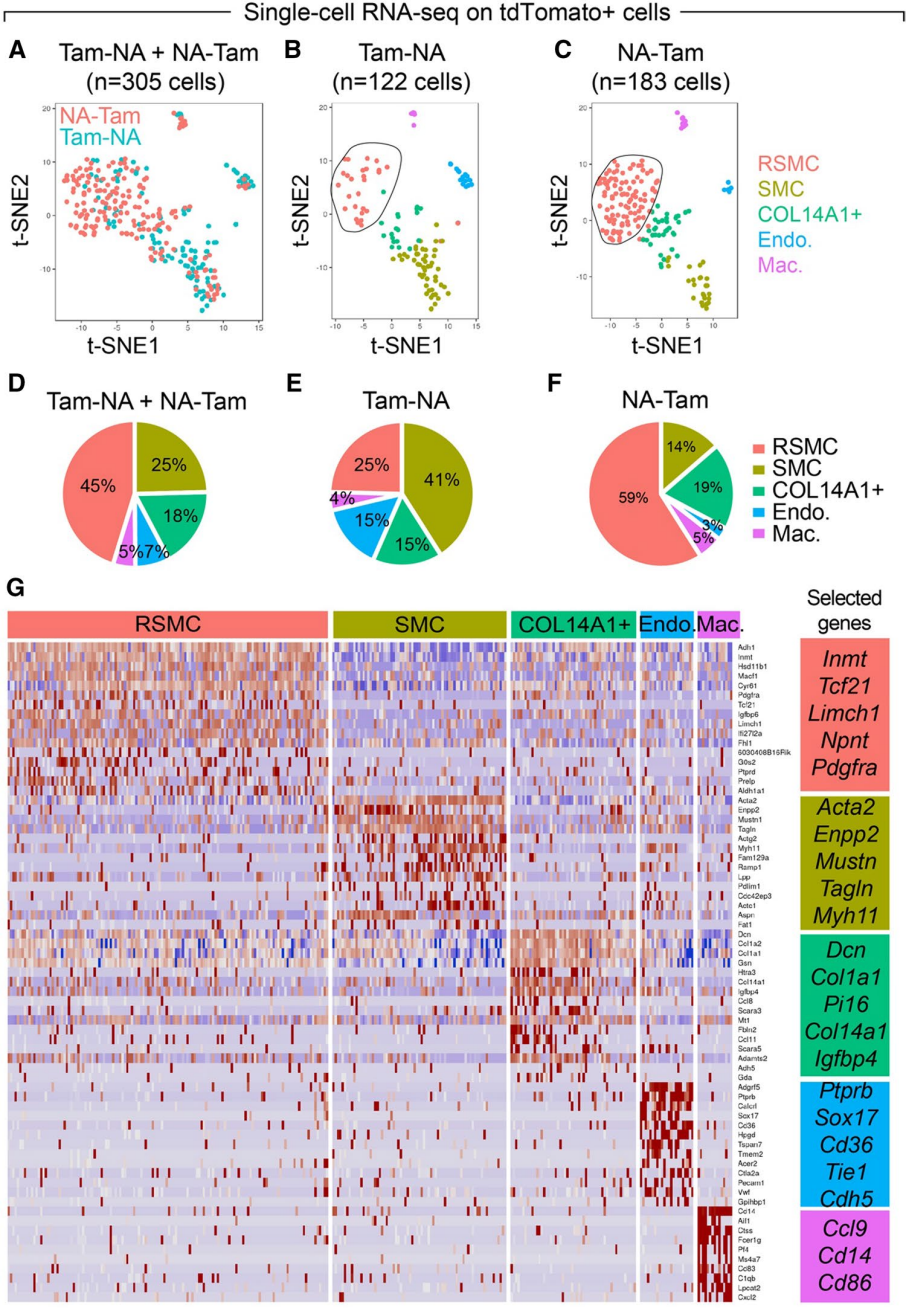
Single-cell RNA sequencing of tdTom+ cells isolated from Tam-NA and NA-Tam *Acta2-Cre-ERT2; tdTomato*^*flox*^ lungs identifies PDGFRα+ RSMCs. **A–C** t-SNE plots showing the distribution of tdTom+ cells among five cellular clusters: RSMC, SMC, COL14A1+ fibroblasts, endothelial cells and macrophages. Note the enrichment of the RSMC cluster in **C**. **D–F** Frequency of each cluster in Tam-NA and NA-Tam lungs. **G** Heatmap showing the top highly enriched genes in the different clusters. Groups of selected genes are shown in the corresponding boxes. *Endo* endothelial cells, *Mac* macrophages, *NA* naphthalene, *RSMC* repair-supportive mesenchymal cells, *SMC* smooth muscle cells, *Tam* tamoxifen. *n = *3 (pooled 3 independent animals) for both Tam-NA and NA-Tam experiments

We next performed scRNA-seq on tdTom+ cells derived from *Acta2-Cre-ERT2; tdTomato*^*flox*^ lungs to delineate the complexity and heterogeneity of these cells, and to identify the RSMC cluster. FACS-sorted tdTom+ cells from Tam-NA (*n = *3 animals) and NA-Tam (*n = *3 animals) were used to dispense single cells onto an ICELL8 chip (Fig. 2). Following library preparation and RNA-seq, quality control yielded 122 cells from the Tam-NA group and 183 cells from the NA-Tam group (Fig. 2A–C). Bioinformatic analysis revealed the presence of five cellular clusters corresponding to SMCs, endothelial cells, macrophages and 2 additional clusters that showed matrix fibroblast signatures, one expressing *Col13a1* and the other one *Col14a1* (Figs. 2A–D, S2A). Similar matrix fibroblast clusters have been previously described [22, 24]. Importantly, the analysis showed a significant emergence of the COL13A1+ matrix fibroblast-like population in NA-Tam lungs compared to Tam-NA lungs (Fig. 2B–D). We have previously reported that deletion of *Ctnnb1* and *Fgf10* in NA-Tam mice led to repair impairment and that *Pdgfra* marks the RSMC population in the NA-Tam condition [14]. In the current study, *Pdgfra* indeed marked the emerging COL13A1+ population corresponding to RSMCs (Figs. 2G and S2A). This cluster expressed typical alveolar fibroblast/lipofibroblast markers such as *Tcf21, Limch1* and *Pdgfra* (Figs. 2G and S2). Comparison between NA-Tam RSMCs and Tam-NA SMC confirmed that *Pdgfra* was among the top genes discriminating between RSMCs and pre-existing SMCs in the context of NA injury (Fig. S2B and Supplementary Table 1).

Differentially expressed genes in COL14A1+ matrix fibroblasts included *Dcn, Col1a1* and *Col14a1* (Figs. 2G and S2A). Cells within the SMC cluster expressed typical SMC markers such as *Acta2, Tagln* and *Myh11* (Figs. 2G and S2A). Differentially expressed genes in the endothelial cluster included *Ptprb*, *Sox17* and *Cdh5* (Figs. 2G and S2A) while in macrophages, genes such as *Ccl9, Cd14* and *Cd86* were differentially expressed (Figs. 2G and S2A).

### The GLI1+ lineage is a source of RSMCs in the lung

The peribronchial and perivascular regions of the lung are populated by hedgehog responsive GLI1+ mesenchymal cells (Fig. S1F). These cells have been shown to contribute to bleomycin-associated myofibroblasts in lung fibrosis [25] and were also shown to be amplified around the airways following NA injury [26]. A proportion of GLI1+ cells is intermingled with ASMCs in the peribronchial space (Fig. S1F). We previously used the *Acta2-Cre-ERT2; tdTomato*^*flox*^ mice to FACS-isolate the RSMC-enriched population (PDGFRα+ tdTom+) and the other mesenchymal populations (SMC-enriched PDGFRα− tdTom+) from NA-Tam animals at d3, d7 and d14 [14] (Fig, S1D). Gene expression analysis showed that *Gli1* is highly expressed in the RSMC-enriched fraction, and its expression peaks at d7 (Fig. S1E). Therefore, we suspected that GLI1+ cells might serve as a source of RSMCs following NA injury.

**Fig. 3:**
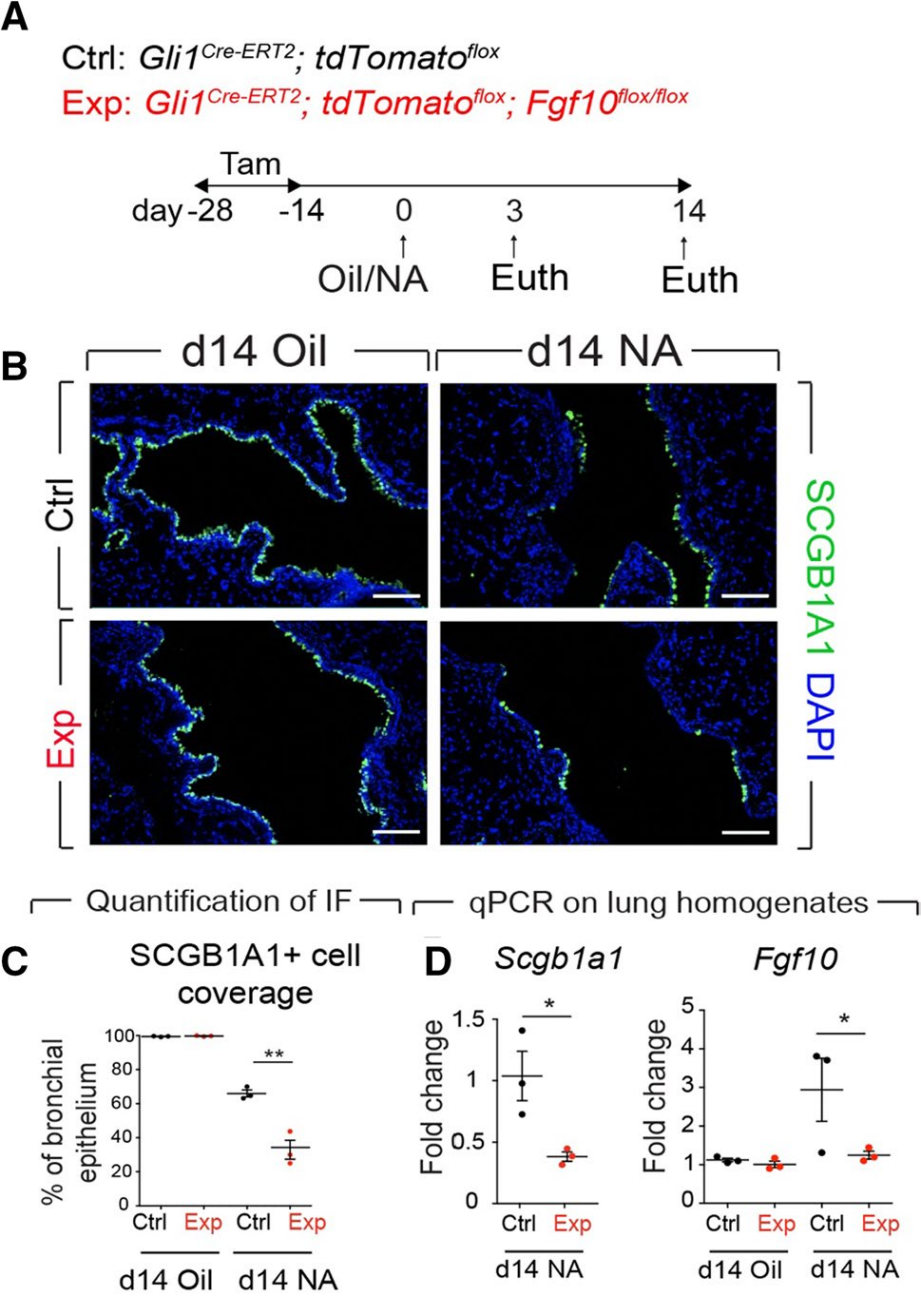
Genetic deletion of *Fgf10* in GLI1+ cells leads to impaired repair in the bronchial epithelium. **A** Experimental setup and timeline of tamoxifen and naphthalene/corn oil treatment. Mice were fed tamoxifen-containing food. **B** Immunofluorescence for SCGB1A1 using control and experimental lungs treated with corn oil or NA and analyzed at d14. **C** Quantification of the immunofluorescence shown in **B** demonstrating decreased abundance of SCGB1A1+ cells in the lungs of experimental mice, indicating impairment of epithelial regeneration. **D** Quantitative real-time PCR using lung homogenates and showing decreased levels of *Scgb1a1* and *Fgf10* gene expression in experimental mice compared to controls. *IF* immunofluorescence, *NA* naphthalene, *Tam* tamoxifen. Scale bars: 100 μm. *n = *3 per group. *P < 0.05, **P < 0.01

To test the hypothesis that pre-existing GLI1+ cells are a source of RSMCs, *Gli1*^*Cre−ERT2*^*; tdTomato*^*flox*^ mice were fed tamoxifen-containing pellets for 2 weeks followed by a 2-week washout period and then treated with NA or Oil (Fig. 3A). This approach allowed labeling of pre-existing GLI1+ cells and studying their fate during epithelial repair after injury. A similar experimental setup was carried out using *Gli1*^*Cre−ERT2*^*; tdTomato*^*flox*^*; Fgf10*^*flox/flox*^ mice to investigate whether FGF10 specifically derived from these cells is required for the repair process (Fig. 3A). Genetic deletion of both *Fgf10* alleles in pre-existing GLI1+ cells significantly impaired club cell replenishment (Fig. 3B, C). Immunofluorescence for KI67 showed a significant decrease in club cell proliferation in experimental (*Gli1*^*Cre−ERT2*^*; tdTomato*^*flox*^*; Fgf10*^*flox/flox*^) vs. control (*Gli1*^*Cre−ERT2*^*; tdTomato*^*flox*^) lungs following NA treatment (Fig. S3). Quantitative real-time PCR using lung homogenates showed significant downregulation of *Scgb1a1* at d14 after NA treatment in experimental mice compared to controls (Fig. 3D). Interestingly, we did not observe a significant drop in *Fgf10* expression in non-injured (Oil condition) experimental vs. control lungs, suggesting that at the resting state, GLI1+ cells are not the main source of *Fgf10* expression in the lung (Fig. 3D). However, following injury, a clear drop in *Fgf10* expression is observed in experimental vs. control lungs fitting with the proposed increase in *Fgf10* expression in the lung during the repair process (Fig. 3D). Of note, we could not detect significant proliferation in the surrounding mesenchyme in the NA vs. Oil-treated control lungs at d14 (Fig. S3), which is in line with the results shown in Fig. S1C, where no change in tdTom+ cells was seen between d7 and d14, thus supporting the notion that RSMCs were no longer expanding after d7.

**Fig. 4:**
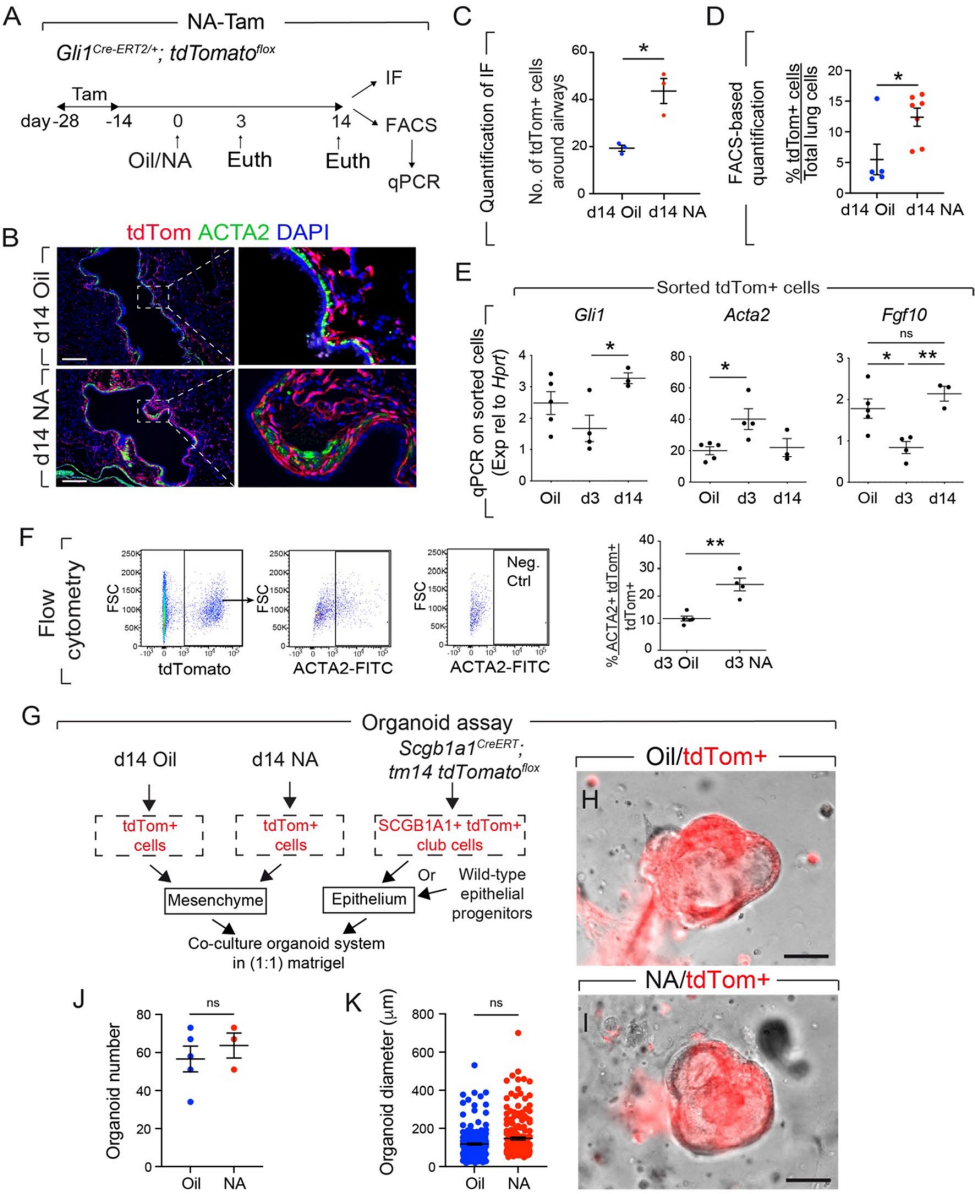
GLI1+ cells give rise to RSMCs and support club cell growth. **A** Experimental setup and timeline of tamoxifen and naphthalene/corn oil treatment. Mice were fed tamoxifen-containing food. **B** Immunofluorescence for ACTA2 using d14 NA and d14 Oil *Gli1*^*Cre−ERT2*^*; tdTomato*^*flox*^ lungs. **C** Quantification of the number of tdTom+ cells around the airways. **D** Quantification of tdTom+ cells by flow cytometry. **E** Expression levels of *Gli1*, *Acta2* and *Fgf10* in tdTom+ cells isolated from Oil, d3 NA and d14 NA lungs. **F** Gating strategy and quantification of cells based on tdTom and ACTA2 expression. **G** Experimental setup for isolating tdTom+ club cells or epithelial progenitors from wild-type lungs, and co-culturing them with tdTom+ cells isolated from oil- or naphthalene-treated *Gli1*^*Cre−ERT2*^*; tdTomato*^*flox*^ lungs. **(H, I)** Representative images of large bronchiolospheres grown from tdTom+ mesenchymal and tdTom+ epithelial cells. **J**, **K** Quantification of organoid number and diameter. *IF* Immunofluorescence, *NA* Naphthalene, *Tam* Tamoxifen, *ns* Not significant. Scale bar: (B, H, I) 100 μm. *n = *4 for d3 and d = 3 for d14; (C) *n = *3; (D) *n = *5 for Oil and *n =* 7 for NA, (E) *n =* 5 for Oil, (F) *n* = 5 for d3 Oil and *n* = 4 for d3 NA ; (J, K) *n = *5 for Oil and *n = *3 for NA. In (K), data from individual organoids are shown. *P < 0.05, **P < 0.01

Examination of Oil-treated *Gli1*^*Cre−ERT2*^*; tdTomato*^*flox*^ mice revealed significant abundance of tdTom+ cells around the airways (Fig. 4A, B). The number of tdTom+ cells was significantly increased in this region upon NA treatment (Figs. 4B, C, S3), as previously described [26]. Such increase was attenuated in experimental lungs vs. controls (Fig. S3). Strikingly, descendants of the GLI1+ lineage were also observed in similar regions as *Acta2-Cre-ERT2*-traced RSMCs (compare Fig. 4B with Fig. 1J, M).

To verify if the increase in *Fgf10* expression (Fig. 3D) is linked to *Fgf10* upregulation in GLI1+ cells, we analyzed *Fgf10* expression in sorted GLI1+ cells. Our results indicate no significant difference in *Fgf10* expression between the d14 group and the Oil group (Fig. 4E). Therefore, the global increase in *Fgf10* expression is likely linked to a quantitative increase in GLI1+ cells. Furthermore, qPCR on tdTom+ cells sorted from Oil- and NA-treated *Gli1*^*Cre−ERT2*^*; tdTomato*^*flox*^ lungs revealed a transient downregulation of *Gli1* at d3 as previously described [26], in parallel to a significant, transient upregulation of *Acta2* at d3 (Fig. 4E). Flow cytometry-based quantification confirmed the acquisition of *Acta2* expression in the GLI1+ lineage upon NA injury (Fig. 4F). Altogether, these data suggest that pre-existing GLI1+ cells 1) transiently acquire *Acta2* expression and give rise to RSMCs during epithelial regeneration (and are therefore captured when ACTA2+ cells are labeled after injury (Fig. 1)), 2) are a source of FGF10 during both steady state and after injury and 3) are massively amplified and recruited to the airways in response to NA injury.

### GLI1+ cells support club cell growth in an in vitro co-culture organoid system

We then opted to test whether GLI1+ cells derived from d14 Oil- and NA-treated lungs possess an intrinsic capacity to support the growth of club cells in a co-culture organoid system. Club cells sorted using uninjured *Scgb1a1*^*CreERT*^*; tm14 tdTomato*^*flox*^ mice or epithelial progenitors sorted from wild-type mice were co-cultured with tdTom+ cells derived from Tam-Oil or Tam-NA *Gli1*^*Cre−ERT2*^*; tdTomato*^*flox*^ mice (Fig. 4G). Representative images of large bronchiolospheres are shown in Fig. 4H, I. Further analysis of these bronchiolospheres indicated a slight trend for increased organoid number and size in the NA vs. Oil condition (Fig. 4J, K), suggesting that the beneficial effect of GLI1+ cells appearing after injury is likely linked to their quantity/recruitment rather than a qualitative change. Additionally, loss of function of *Fgf10* in GLI1+ cells led to decreased organoid size compared to controls (Fig. S4).

### Single-cell RNA-seq of GLI1+ cells suggests that alveolar fibroblasts, co-expressing *Gli1* and *Fgf10*, converge on the RSMC lineage

**Fig. 5:**
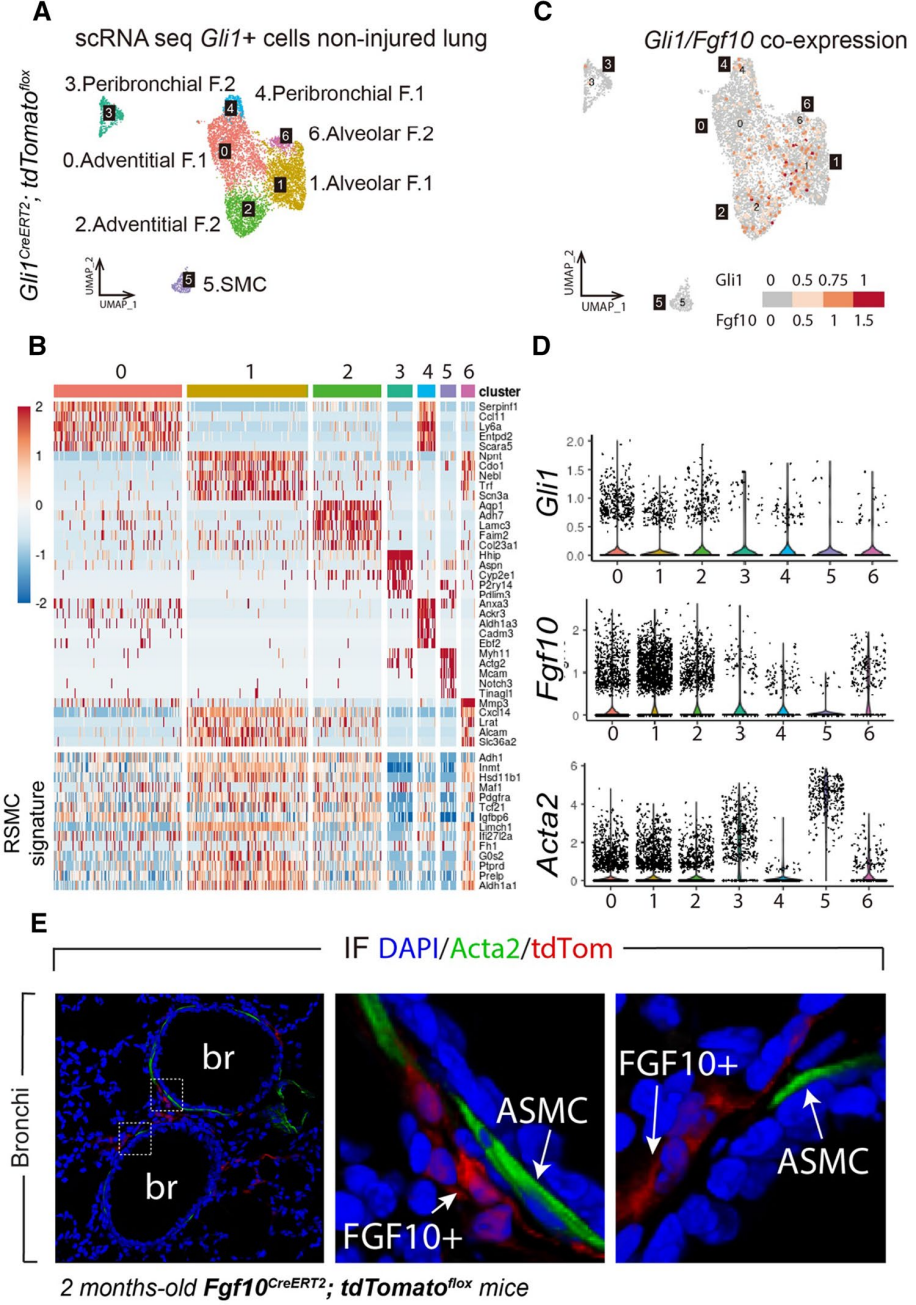
scRNA-seq analysis of GLI1+ cells confirms the presence of an RSMC cluster. **(A)** UMAP clustering of GLI1+ cells using uninjured *Gli1*^*Cre−ERT2*^*; tdTomato*^*flox*^ lungs. **(B)** Upper panel: Heatmap showing the top highly enriched genes in the different clusters. Lower panel: Heatmap showing the top enriched RSMC signature genes in different clusters. **(C)** Analysis of the co-expression of *Gli1* and *Fgf10* in different clusters. **(D)** *Gli1, Fgf10* and *Acta2* expression throughout the different clusters. **(E)** Immunofluorescence images of *Fgf10*^*Cre−ERT*^*; tdTomato*^*flox*^ lungs showing FGF10+ cells around the bronchi and distinct from ASMCs. *ASMC* airway smooth muscle cells, *br* bronchus, *IF* immunofluorescence

The organoid assay suggested that GLI1+ cells have an intrinsic club cell-supportive potential even in the absence of injury (Fig. 4G-K). We therefore explored the heterogeneity of GLI1+ cells in non-injured lungs using scRNA-seq (Fig. 5). UMAP analysis showed the presence of seven clusters (Fig. 5A). Cluster 1 corresponds to alveolar fibroblasts, while cluster 6 represents a subcluster of alveolar fibroblasts expressing high levels of *Mmp3* (Fig. 5A, B). Clusters 0 and 2 contain two adventitial fibroblast subsets. Clusters 3 and 4 include peribronchial fibroblasts, while cluster 5 corresponds to SMCs. Examination of the RSMC transcriptomic signature defined in Fig. 2 shows that most of the signature genes are contained mostly in alveolar fibroblasts (clusters 1 and 6), and to a less extent in adventitial fibroblasts (Fig. 5B lower panel). Interestingly, cluster 5 (SMCs) did not display significant RSMC signature, thus supporting the notion that RSMCs are distinct from SMCs. Given the important role played by *Fgf10* expressed by GLI1+ cells in the repair process and the fact that *Fgf10* is not significantly upregulated in these cells after NA injury, we reasoned that GLI1+ FGF10+ cells exist in the non-injured lung. This is rather counter-intuitive as *Gli1*, a downstream target of sonic hedgehog signaling, and *Fgf10* expression are thought to be mutually exclusive especially during lung development [27]. Confirming our hypothesis, these cells were found in alveolar fibroblasts and adventitial fibroblasts but completely absent in the SMCs (Fig. 5C). Quantification of the expression of *Gli1*, *Fgf10* and *Acta2* confirmed that alveolar fibroblasts represent the main cluster displaying high levels of *Fgf10* as well as moderate levels of *Gli1* and *Acta2* (Fig. 5D). Using our novel *Fgf10*^*Cre−ERT2*^ mice recently reported [28], we also examined the localization of FGF10+ cells in non-injured lungs. In addition to being located in the alveolar region [28], FGF10+ cells were also observed around the bronchi and distinct from ASMCs (Fig. 5E), similarly to what was observed for GLI1+ cells (Fig. S1F).

## Discussion

In this study, we demonstrated that GLI1+ cells are a source of RSMCs during airway epithelial regeneration. Lineage tracing of ACTA2+ cells (using the *Acta2-Cre-ERT2* driver line) labeled either before (Tam-NA) or after (NA-Tam) injury indicates that these cells appear mostly following injury. Nevertheless, lineage-tracing experiments using the *Gli1*^*Cre−ERT2*^ driver line indicate that RSMCs also arise from pre-existing GLI1+ cells that acquire *Acta2* expression and accumulate in the peribronchial region. Deletion of *Fgf10* in GLI1+ cells leads to impaired repair. Using bronchiolosphere assays, we show that GLI1+ cells isolated from either non-injured or NA-treated lungs similarly support club cell growth in vitro. Overall, our data indicate that GLI1+ cell-derived RSMCs produce FGF10 that is required for the repair process after NA injury. As such, RSMCs represent a privileged mesenchymal niche for club cell progenitors.

### The lineage map of the lung mesenchyme is still poorly understood

Despite extensive research, the lineage map of the lung mesenchyme is still poorly understood, with the best-characterized cell types being ASMCs/VSMCs and pericytes, and, to a less extent, alveolar fibroblasts (mostly during neonatal stages) and myofibroblasts. The perivascular region of the lung is populated by GLI1+ mesenchymal cells that have been proposed to constitute a subpopulation of PDGFRβ+ pericytes [25]. These cells have been shown to possess mesenchymal stem cell (MSC)-like characteristics such as trilineage differentiation potential in vitro (giving rise to adipocytes, osteoblasts and chondrocytes) and also represent a repertoire of myofibroblast progenitors in fibrotic disease [25, 29]. On the other hand, the peribronchial domain of the postnatal mouse lung consists of cartilaginous rings (around main-stem bronchi), ASMCs (around conducting airways) and other poorly characterized mesenchymal cells, some of which also express *Gli1*.

### RSMCs are a component of the niche required for activating club cell progenitors

**Fig. 6:**
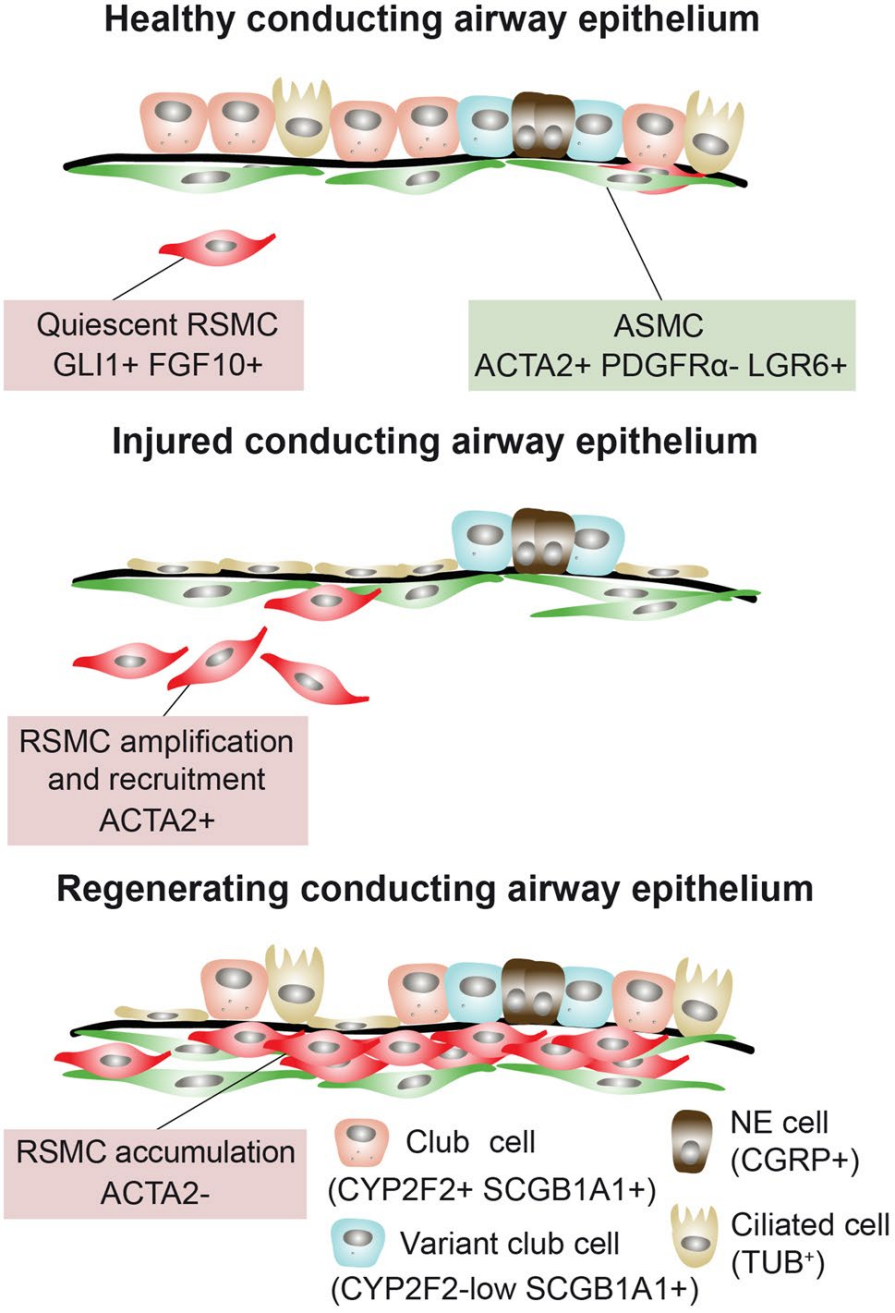
Model summarizing the role of RSMCs as a niche that facilitates epithelial regeneration after naphthalene injury. GLI1+ cells are distinct from ASMCs. After naphthalene injury, GLI1+ cells transiently acquire *Acta2* expression and get amplified, giving rise to RSMCs and contributing to epithelial regeneration by producing FGF10. Pre-existing ASMCs have also been shown to mediate the repair process via a WNT-FGF10-mediated mechanism [12, 13]. *CGRP* calcitonin gene-related peptide, *CYP2F2* cytochrome P450 2F2, *NE* neuroendocrine, *TUB* tubulin

In this study, we demonstrate the complexity of the mesenchymal niche in the mature lung and identify GLI1+ cell-derived RSMCs as a component of the niche required for activating club cell progenitors. GLI1+ cells are amplified, transiently acquire *Acta2* expression and contribute to the repair process by producing FGF10 (Fig. 6). We previously published side-by-side comparison of PDGFRα+ tdTom+ cells (RSMC-enriched population) and PDGFRα− tdTom+ cells (SMC-enriched population), indicating that RSMCs display higher WNT signaling activation (using *Axin2* mRNA levels as readout), higher *Fgf10* expression and greater potential in supporting club cell growth in an in vitro organoid system [14]. However, one limitation was that the PDGFRα− tdTom+ population contains a mixture of *bona fide* ASMCs/VSMCs and other subsets such as matrix fibroblasts rather than a pure population of ASMCs, and this might reduce the colony-forming efficiency and consequently bronchiolosphere number and size. Moreover, we could not exclude the possibility that parenchymal lineage-labeled cells (such as alveolar fibroblasts) also contribute to repair in the conducting airway epithelium. Our data strongly indicate that GLI1+ cells, which are normally found in the peribronchial, perivascular and alveolar regions, serve as a source of FGF10+ RSMCs in the lung.

### How different are RMSCs from the resident mesenchymal cell (rMC) niche?

Resident mesenchymal cells supporting the growth of alveolar stem cells were further characterized [30]. Using flow cytometry to isolate cells as well as the alveolosphere model, CD31− CD45− EpCAM− cells (defined as rMC) could be subdivided into SCA1+ and SCA1−. Only SCA1+ rMCs displayed niche activity for AT2 stem cells. Using reporter lines for *Fgf10* expression, these cells were further subdivided into SCA1+ FGF10+ rMCs and SCA1+ FGF10− rMCs, with the FGF10+ subset displaying the niche activity. These cells are also positive for PDGFRα.

We also recently used scRNA-seq to further characterize the different subpopulations present in SCA1+ rMCs [31]. We identified eighteen different clusters. Among them, five different clusters matched different subsets of lipofibroblasts and specifically expressed *Fgf10*, *Fgf7* and *Pdgfra*. These clusters could also be differentiated via the level of *Col13a1*, *Col1a1* and *Col14a1* expression. One of these five clusters expresses high levels of *Col13a1*, *Fgf10*, *Limch1*, *inmt*, *Npnt* and *Pdgfra*, and could therefore be very similar to RSMCs. Interestingly, this cluster expresses lower levels of *Sca1* compared to the other clusters. This puzzling similarity between RSMCs around the bronchi and rMC niche cells in the alveolar region raises the possibility that rMC niche cells could be recruited from the adjacent alveolar region surrounding the bronchi. Further investigation using high-resolution imaging and more specific lineage tracing using the Dre/Cre technology should allow answering this important question.

## Supplementary Information

Below is the link to the electronic supplementary material.

Supplementary file1 Supplementary Table 1. Top differentially expressed genes in tdTom+ cells between NA-Tam RSMCs and Tam-NA SMCs. (CSV 108 kb)

Supplementary file2 (DOCX 2104 kb)

## Data Availability

Primary data can be made available upon reasonable request. Single-cell data have been deposited on gene expression omnibus (GEO) GSE215094.
