# Supplementary material for: GLI1+ cells are a source of repair-supportive mesenchymal cells (RSMCs) during airway epithelial regeneration
Source: Cell Mol Life Sci. 2022 Nov 5;79(11):581. doi: 10.1007/s00018-022-04599-2 (PMC9636301; doi:10.1007/s00018-022-04599-2)
Supplement: Supplementary file 2 — Supplementary file2 (DOCX 2104 kb) [file 18_2022_4599_MOESM2_ESM.docx]

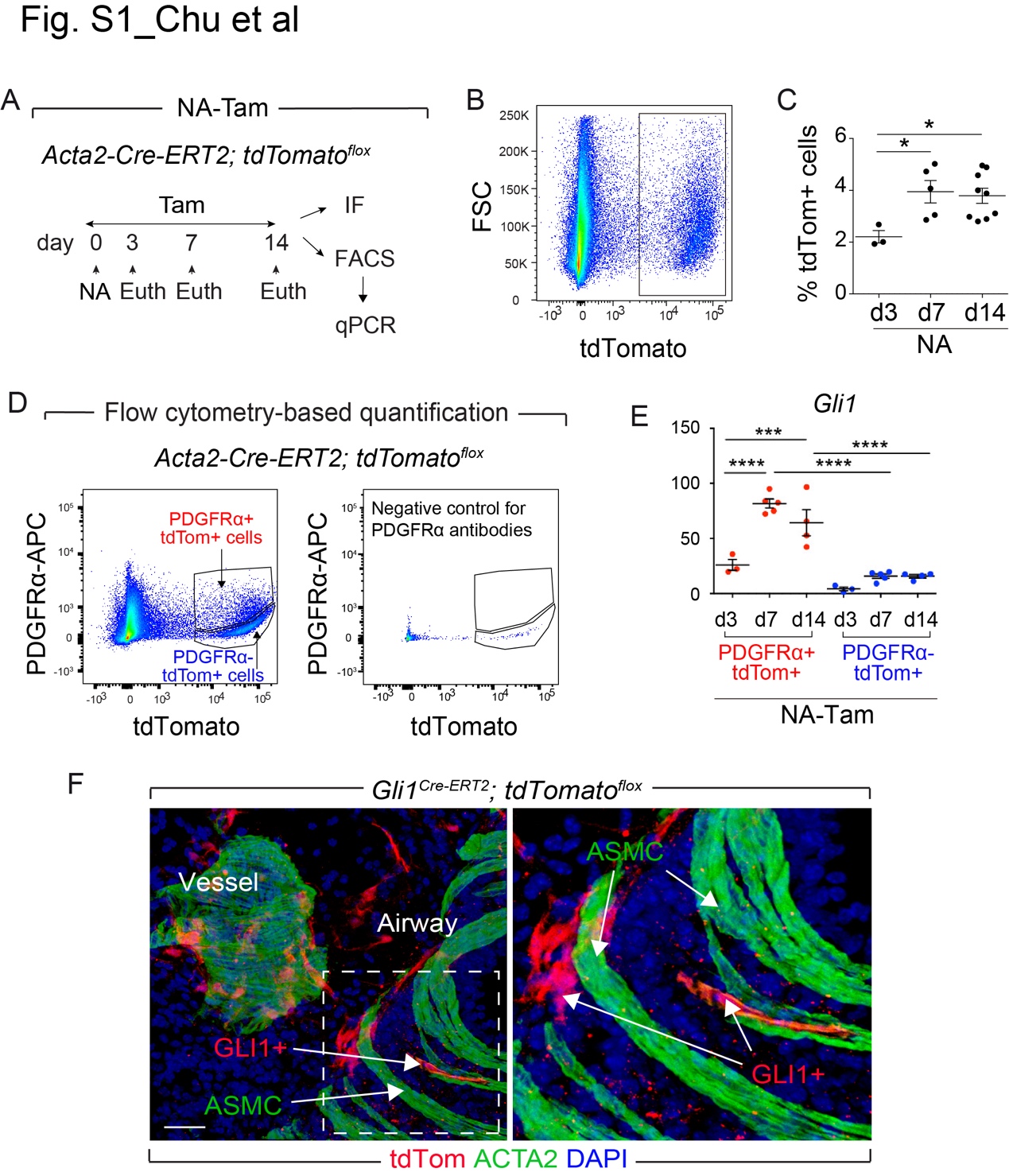


**Fig. S1** RSMCs are enriched with *Gli1* expression. **(A)** Experimental setup and timeline of tamoxifen and naphthalene treatment. Mice were fed tamoxifen-containing food. **(B, C)** Flow cytometric analysis demonstrating an increase in the percentage of tdTom+ cells from d3 to d7. Note the lack of change in the abundance of these cells between d7 and d14 (the d14 data are replicated from Fig. 1O). **(D)** Gating strategy for isolating RSMC-enriched (PDGFRα+ tdTom+) vs. PDGFRα- tdTom+ from *Acta2-Cre-ERT2; tdTomato^flox^* mice following NA injury. **(E)** Expression levels of *Gli1* as determined by qPCR in PDGFRα+ tdTom+ cells vs. PDGFRα- tdTom+ cells. **(F)** Three-dimensional (3D) reconstruction of a *Gli1^Cre-ERT2^; tdTomato^flox^* lung section stained with anti-ACTA2 antibodies. Note the presence of tdTom+ cells intermingled with ASMCs. The area in the box is magnified. *IF* immunofluorescence, *NA* naphthalene, *Tam* tamoxifen. Scale bar: F: 25 μm. (C) *n* = 3 for d3, *n* = 5 for d7 and *n* = 9 for d14; (E) *n* = 3 for d3, *n* = 5 for d7 and *n* = 4 for d14. * P<0.05, *** P<0.001, **** P<0.0001.


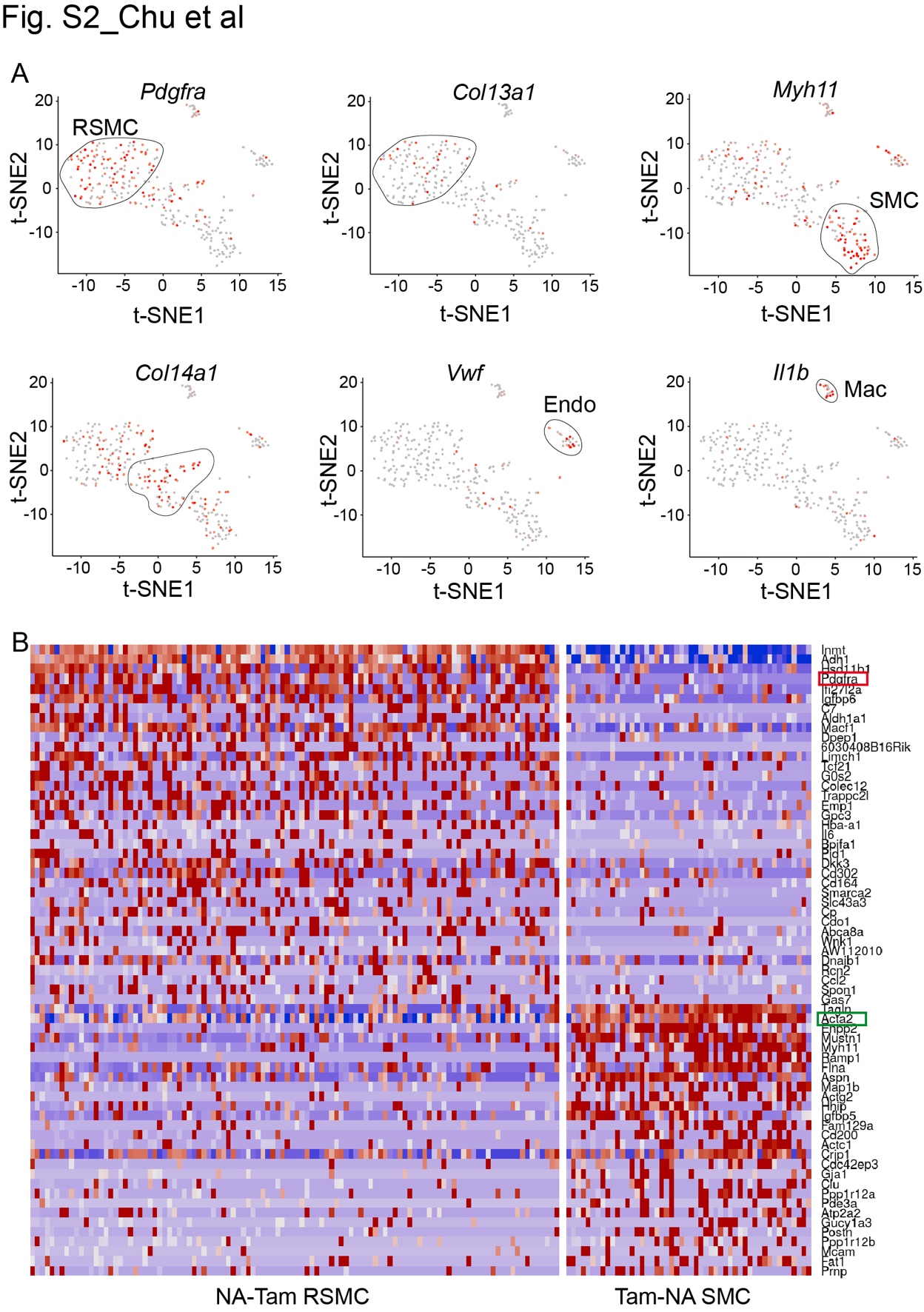


**Fig. S2** scRNA-seq identified top regulated genes between RSMCs and SMCs. **(A)** t-SNE plots for *Pdgfra*, *Col13a1*, *Myh11*, *Col14a1*, *Vwf* and *Il1b.* (**B**) Heatmap for the top differentially expressed genes between NA-Tam RSMCs and Tam-NA SMCs. Note the differential expression of *Pdgfra* (Red box) and *Acta2* (Green box).


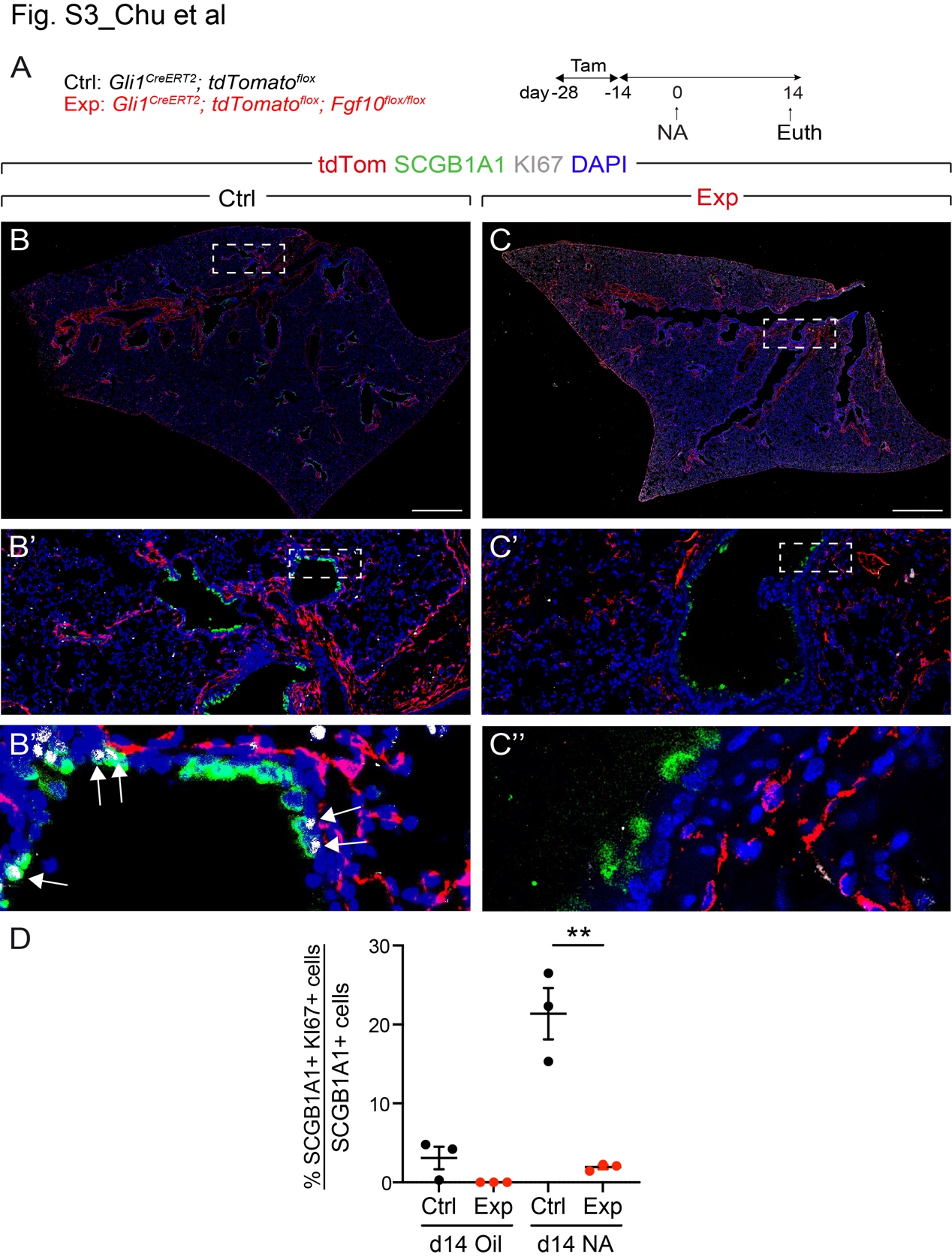


**Fig. S3** Analysis of proliferation in control and experimental lungs. **(A)** Experimental setup and timeline of tamoxifen and naphthalene treatment. Mice were fed tamoxifen-containing food. **(B-B’’)** Immunofluorescence for tdTom, SCGB1A1 and KI67 in control (Ctrl) lungs. **(C-C’’)** Immunofluorescence for tdTom, SCGB1A1 and KI67 in experimental (Exp) lungs. **(D)** Quantification of proliferating club cells. Scale bars: 1 mm. *n* = 3. ** P<0.01.


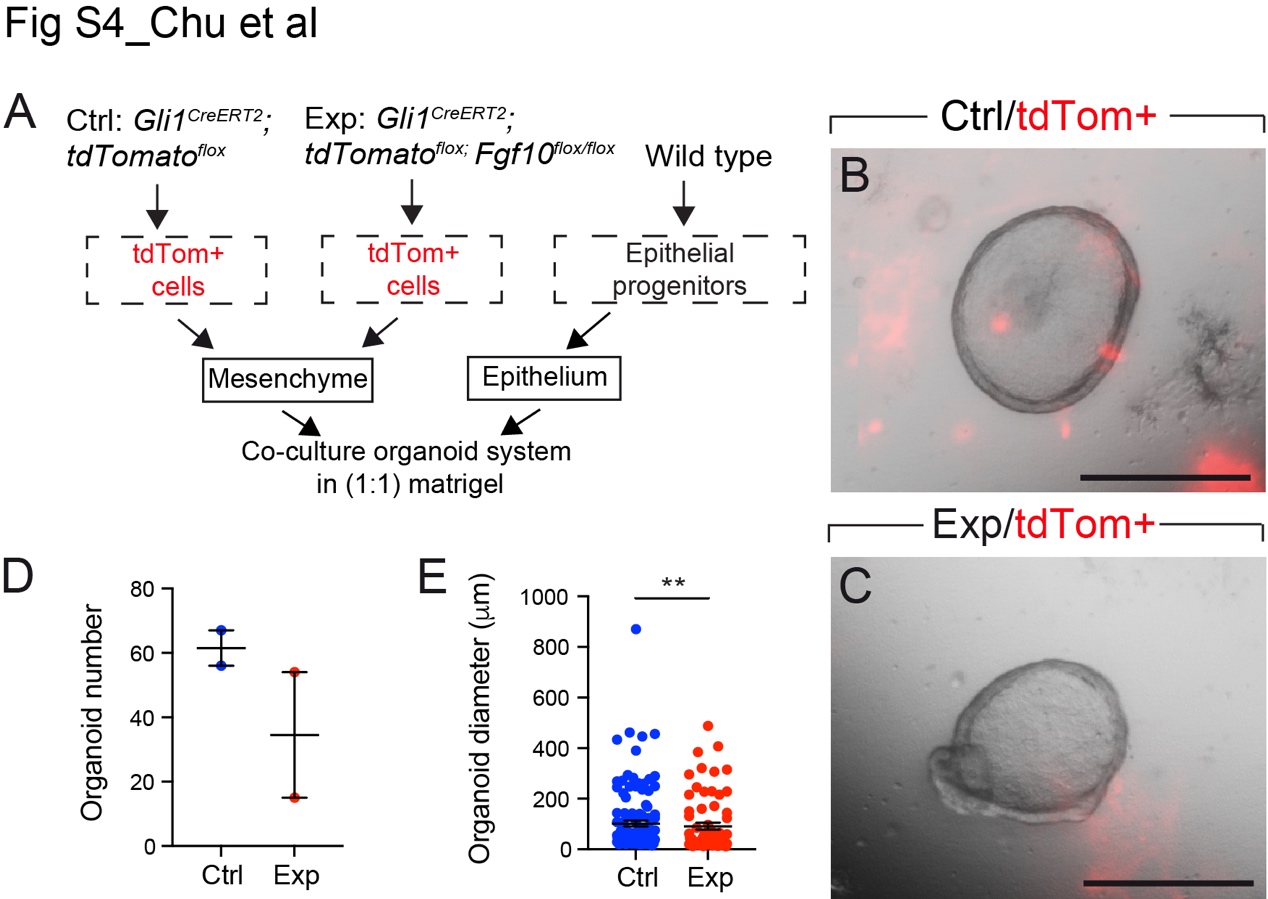


**Fig. S4** Loss of function of *Fgf10* in GLI1+ cells impairs their supportive potential *in vitro*. **(A)** Experimental setup for isolating epithelial progenitors from wild-type lungs and co-culturing them with tdTom+ cells isolated from control (Ctrl) *Gli1^Cre-ERT2^; tdTomato^flox^* or experimental (Exp) *Gli1^Cre-ERT2^; tdTomato^flox^; Fgf10^flox/flox^* lungs. **(B, C)** Representative images of organoids grown from each condition. **(D, E)** Quantification of organoid number and diameter. Scale bars: 100 μm. (D) *n* = 2. In (E), data from individual organoids are shown. ** P<0.01.
